# Supplementary material for: Comparing infiltration rates in soils managed with conventional and alternative farming methods: A meta-analysis
Source: PLoS One. 2019 Sep 19;14(9):e0215702. doi: 10.1371/journal.pone.0215702 (PMC6752860; doi:10.1371/journal.pone.0215702)
Supplement: S1 File — Table A. Description of experiments included in the meta-analysis database Fig A. Response of infiltration rates to sub-categories of crop rotation experiments (n = number of paired comparisons per group). (a) Paired comparisons grouped by the number of crops included in the experimental treatment rotation. (b) Paired comparisons grouped by the main crop in the control treatment. Note that this does not include Govaerts et al. (2007) which reported maize monoculture and wheat monoculture separately. Fig B. Percent change in infiltration rates for perennial experiments. Paired comparisons grouped by the three types of included treatments: agroforestry, perennial grasses, and managed forestry (n = number of paired comparisons per group). Fig C. Response of infiltration rates for crop and livestock (cropland grazing) experiments. Paired comparisons grouped by the presence of annual crops or pasture in control and experimental treatments (n = number of paired comparisons per group). Fig D. Experiment locations for each of the different agricultural practices included in the analysis. (1) no-till, (2) cover crops, (3) crop rotation, (4) crop and livestock, (5) perennials. Maps generated with ESRI ArcGIS ver. 10.4 (http://www.esri.com). Fig E. Natural log response ratio by practice across the range of aridity indices. Response ratios above zero (solid line) represent a positive effect of the practices on infiltration rates. Smoothed means are represented in blue and gray. Aridity index values greater than 0.65 (dashed line) represent regions considered to have more humid climates. Fig F. Natural log response ratio by practice category across the range of (1) sand and (2) clay contents. Smoothed means are represented in blue and gray. Dashed lines represent the broad groups of sand and clay (>50% sand, >30% clay) that were used for the fixed effects analysis in the cover crop and no-till experiments. As a result of data limitations, this figure does not represent some expe [file pone.0215702.s002.docx]

**Supporting Information**

**Comparing infiltration rates in soils managed with conventional and alternative farming methods: a meta-analysis**

**Andrea D. Basche^1*^ and Marcia S. DeLonge^2^**

1. Department of Agronomy and Horticulture, University of Nebraska-Lincoln, Lincoln, NE, U.S.A.
2. Food & Environment Program, Union of Concerned Scientists, Oakland, CA, U.S.A.

* Corresponding author:

E-mail: abasche2@unl.edu

Keywords: infiltration rates; meta-analysis; no-till; cover crops; crop rotations; perennial crops; crops and livestock; rainfall variability; climate change adaptation

**Table A. Description of experiments included in the meta-analysis database**

| State/Region, Country | Category | Main cropping system and description of experimental treatment | Control treatment | Reference |
| --- | --- | --- | --- | --- |
| Denmark | cover crop, no-till | barley with radish cover crop, no-till | no cover crop, conventional tillage, reduced tillage | Abdollahi et. al 2014 |
| Texas, USA | crop rotation, no-till | sorghum-wheat | continuous sorghum, reduced tillage | Alemu et al. 1997 |
| Yurimaguas, Peru | crop and livestock | trees, pasture, maize and livestock grazing | trees and pasture^ | Arevalo et al. 1998 |
| British Columbia, Canada | no-till | continuous barley | conventional tillage | Arshad et al. 1999 |
| Central Mexico | cover crop, no-till | no-till, maize with vetch or oat cover crop | conventional tillage, maize without a cover crop | Astier et al. 2006 |
| Uttarakhand, India | no-till | rice-wheat no-till | conventional tillage | Bajpai and Tripathi 2000 |
| Santa Cruz, Bolivia | no-till | wheat-soybean-sunflower no-till | conventional tillage, reduced tillage | Barber et al. 1996 |
| Texas, USA | no-till | wheat-sorghum-fallow no-till | reduced tillage | Baumhardt and Jones 2002 |
| Texas, USA | crop rotation | wheat-sorghum | continuous wheat | Baumhardt et al. 2012 |
| Uttar Pradesh, India | no-till | rice-wheat no-till | conventional tillage, reduced tillage | Bazaya et al. 2009 |
| NSW, Australia | crop and livestock | wheat or canola with sheep grazing | canola and wheat only | Bell et al. 2011 |
| Iowa, USA | perennial | silver maple, grass filter, switchgrass, grazed pasture# | maize-soybean* | Bharati et al 2002 |
| Uttarakhand, India | no-till | rice-wheat no-till | conventional tillage | Bhattacharyya et al 2008 |
| Kansas, USA | crop rotation | sorghum-wheat-soybean | continuous sorghum | Blanco Canqui et al 2010 |
| Kansas, USA | cover crop | winter wheat-grain sorghum with sunnhemp and late maturing soybean cover crops | winter wheat-grain sorghum with no cover | Blanco Canqui et al 2011 |
| Georgia, USA | no-till | sorghum-soybean no-till | conventional tillage, reduced tillage | Bruce et al. 1990 |
| Georgia, USA | cover crop and no-till | soybean-grain sorghum-crimson clover no-till~ | conventional tillage soybean-grain sorghum-fallow | Bruce et al. 1992 |
| Southern Malawi | perennial | maize with sesbania, gliricidia, leucaena, acacia intercrops | continuous maize | Chirwa et al. 2003 |
| Oklahoma, USA | no-till | continuous wheat no-till | conventional tillage | Dao 1993 |
| Parana, Brazil | no-till | wheat-soybean no-till | conventional tillage | de Moraes et al 2016 |
| Norhern Pampean Region, Argentina | crop and livestock | maize-soybean and grass alfalfa pasture rotation with cattle grazing | maize-soybean only | Fernandez et al. 2015 |
| Kampala, Uganda | cover crop | maize-bean with crotaleria green manure | maize-bean only | Fischler et al. 1999 |
| California, USA | cover crop | almond orchard with bromegrass or clover cover crop, tomato with oat or vetch cover crop | orchard no cover crop, tomato no cover crop | Folorunso et al 1992 |
| Ibadan, Nigeria | no-till | continuous maize no-till | reduced tillage | Franzen et al. 1994 |
| Georgia, USA | crop and livestock | varied intensity cattle grazing on forage grass | hayed forage grass^ | Franzluebbers et al. 2012 |
| Georgia, USA | no-till | sorghum-maize-cereal rye cover crop no-till, winter wheat-pearl millett cover crop no-till | conventional tillage | Franzluebbers et al. 2008 |
| Meerut, India | no-till | rice-wheat no-till | conventional tillage, reduced tillage | Gangwar et al. 2006 |
| Central Indus Plain, India | cover crop | rice-wheat-sesbania green manure | rice-wheat without cover crop | Ghafoor et al. 2012 |
| Meghalaya, India | perennial | perennial grasses cut for livestock feed | continuous cultivation annual crops | Ghosh et al. 2009 |
| Southern Nigeria | no-till | maize-maize-cowpea no-till | conventional tillage | Ghuman and Lal 1992 |
| Southwest Spain | no-till | oat-triticale-vetch-brassica no-till | conventional tillage | Gomez-Paccard et al. 2015 |
| Central Mexico | crop rotation, no-till | maize-wheat (crop rotation), no-till | continuous maize and continuous wheat (crop rotation)*, conventional tillage | Govaerts et al. 2007 |
| Erzurum, Turkey | no-till | wheat-vetch no till | conventional tillage, reduced tillage | Gozubuyuk et al. 2014 |
| California, USA | cover crop | grape vineyard with bromegrass cover crop | grape vineyard no cover crop | Gulick et al 1994 |
| Dodoma, Tanzania | no-till | sorghum no till | conventional tillage, reduced tillage | Guzha 2004 |
| Shaanxi province, China | no-till | winter wheat no-till (with residue retention)~ | conventional tillage | He et al. 2009 |
| Uttar Pradesh, India | no-till | rice-wheat no till | conventional tillage | Jat et al. 2009 |
| Uttar Pradesh, India | no-till | maize-wheat no till | conventional tillage | Jat et al. 2013 |
| Punjab province, Pakistan | cover crop | wheat-cotton with a jantar green manure | no cover crop | Kahlown and Azam 2003 |
| Iowa, USA | cover crop | maize-soybean-winter rye cover crop | maize-soybean no cover crop | Kaspar et al. 2001 |
| Ibadan, Nigeria | no-till | maize-cowpea-soybean no-till | conventional tillage | Kayombo et al. 1991 |
| Southern Ethiopia | perennial | maize, forestry and cattle grazing# | continuous maize with tillage | Ketema and Yimer 2014 |
| West Bengal, India | no-till | peanut no-till | conventional tillage, reduced tillage | Khan 1984 |
| Ohio, USA | crop rotation, no-till | maize-soybean, no-till | continuous maize, reduced tillage | Kumar et al. 2012 |
| Meghalaya, India | no-till | groundnut-rapeseed no-till | conventional tillage | Kuotsu et al 2014 |
| South-Limbourg, Netherlands | cover crop | maize silage with winter rye or summer barley cover crops | no cover crop | Kwaad and Van Milligan 1991 |
| Ibadan, Nigeria | cover crop | maize-cowpea-pigeon pea-cassava-soybean with cover crops | no cover crop | Lal et al. 1978 |
| Ibadan, Nigeria | no-till | continuous maize | moldboard plow, ridge till, disc plow | Lal 1997 |
| Ohio, USA | no-till | maize-soybean no-till | reduced tillage | Lal et al. 1989 |
| Rajasthan, India | no-till | sorghum interseeded with green gram | conventional tillage, reduced tillage | Laddha and Totawat 1997 |
| Georgia, USA | perennial | long leaf pine, planted pine | corn-soybean conventional tillage | Levi et al. 2010 |
| North Dakota, USA | perennial, no-till | grazed pasture (perennial), spring wheat-winter wheat no-till (no-till)~ | annual cropping sequence with no grazing (perennial), conventional tillage with spring wheat-fallow (no-till) | Liebig et al. 2004 |
| North Dakota, USA | crop and livestock, perennial | oat/pea-triticale/sweet clover-maize no till with grazing animals (crop and livestock), western wheatgrass pasture cut for forage (perennial) | hayed pastured grass (crop and livestock)*^, oat/pea-triticale/sweet clover-maize no till with grazing animals (perennial) | Liebig et al. 2011 |
| Pulawy, Poland | no-till | maize-spring barley-winter rape-winter wheat-faba bean no-till | conventional tillage, reduced tillage | Lipiec 2006 |
| Mississippi, USA | no-till, cover crop | cotton-soybean no-till with rye or vetch cover crop | no cover crop, reduced tillage | Locke et al. 2012 |
| Iowa, USA | no-till | maize-soybean no-till | conventional tillage, reduced tillage | Logsdon et al. 1992 |
| Punjab province, Pakistan | cover crop | cotton-wheat with berseem grown as a green manure | cotton-wheat no cover crop | Mahmood-ul-Hassan et al 2013 |
| Tel Hadya, Syria | crop and livestock | wheat-lentil-chickpea-vetch-watermelon with livestock | crops only no grazing | Masri and Ryan 2006 |
| Georgia, USA | cover crop | grain sorghum with vetch or wheat cover crop | sorghum fallow no cover crop | McVay et al. 1989 |
| New York, USA | no-till | maize no-till | plow tillage | Moebuis Clune 2008 |
| Uttar Pradesh, India | no-till | rice no-till | conventional tillage | Naresh et al. 2014 |
| Kpong, Ghana | cover crop | maize with stylosanthes guianesis, mucuna pruriens, and mimosa invisa cover crops | maize no cover crop | Nyalemegbe et al. 2011 |
| Harare, Zimbabwe | crop rotation, no-till | maize-sesbania and maize-A. angustissima (crop rotation), no-till | continuous maize (crop rotation), conventional tillage | Nyamadzawo et al. 2003, Nyamadzawo et al. 2008 |
| Seville province, Spain | no-till | wheat-sunflower no-till | conventional tillage, reduced tillage | Pelegrin et al. 1990 |
| Multiple North America locations: South Dakota, North Dakota, Nebraska, Saskatchewan | crop rotation, no-till | maize-soybean-spring wheat-alfalfa (crop rotation), maize-soybean-sorghum-oat/clover (crop rotation), spring wheat-lentil (crop rotation), spring wheat-pea no-till | continuous maize (crop rotation x2 locations), spring wheat only (crop rotation), spring wheat-pea conventional tillage | Pikul et al. 2005 |
| Western Australia | crop and livestock | pasture grazed with sheep | hayed pasture^ | Proffitt et. al 1995 |
| Punjab province, India | no-till | soybean-wheat no-till | conventional tillage | Ram et al. 2013 |
| central Mozambique | crop rotation | maize-pigeonpea intercrop | continuous maize | Rusinamhodzi et al 2012 |
| Entre Rios province, Argentina | no-till | wheat-maize-soybean no-till | reduced tillage | Sasal et al. 2006 |
| Uttarakhand, India | no-till | rice-wheat no-till | conventional tillage, reduced tillage* | Sharma et al. 2005 |
| Uttarakhand, India | cover crop | maize-wheat with sunnhemp, leucaena green manures | maize-wheat no cover crop | Sharma et al. 2010 |
| Jammu and Kashmir, India | no-till | maize-wheat no-till | conventional tillage, reduced tillage | Sharma et al. 2011 |
| Alaska, USA | no-till | barley no-till | conventional tillage, reduced tillage | Sharratt et al. 2006 |
| Edmonton, Canada | no-till | continuous barley no-till | conventional tillage | Singh et al. 1996 |
| Punjab province, India | cover crop | rice-wheat with sesbania aculeata green manure | rice-wheat without cover crop | Singh et al. 2007 |
| Uttar Pradesh, India | no-till | rice-maize no-till | conventional tillage | Singh et al. 2016 |
| NSW, Australia | no-till | barley-oats no-till | conventional tillage | So et. al 2009 |
| Hawkes Bay, New Zealand | no-till, cover crop | summer-winter vegetables (tomato, broad bean, sweet maize, cauliflower, sweet pepper, broccoli) with annual ryegrass cover crop (cover crop), no-till summer-winter vegetables | conventional tillage, no cover crop | Springett et al. 1992 |
| Maryland, USA | cover crop | maize with rye cover crop | no cover crop | Steele et al. 2012 |
| Nkhotakota and Dowa districts, Malawi | crop rotation, no-till | maize-cassava-pigeon pea (crop rotation), no-till | continuous maize (crop rotation). conventional tillage | TerAvest et al 2015 |
| central Greece | cover crop | cotton with vicia sativa or durum wheat cover crop | no cover crop | Terzoudi et al. 2007 |
| Monze, Zambia | crop rotation | maize-cotton, maize-sunnhemp | continuous maize | Theifelder and Wall 2010 |
| Queensland, Australia | crop rotation | lucerne, medic annual pasture and wheat# | continuous wheat | Thomas et al. 2009 |
| Queensland, Australia | no-till | sorghum-wheat no-till | conventional tillage, reduced tillage | Thorburn et al. 1992 |
| Uttarakhand, India | no-till | rice-wheat | conventional tillage | Tripathi et al. 2007 |
| Punjab province, India | cover crop | rice-wheat- Sesbania green manure | no cover crop | Walia et al. 2010 |
| Shaanxi province, China | perennial | alley cropping with walnut-wheat, monoculture walnut | continuous wheat | Wang et al. 2015 |
| Ibadan, Nigeria | cover crop | maize-cowpea-cassava with cover crops | no cover crop | Wilson and Lal 1982 |
| Haryana, India | no-till | rice-wheat no-till | conventional tillage | Yaduvanshi and Sharma 2014 |
| *Averaged controls | | | | |
| #Experimental treatment confounded by livestock | | | | |
| ~He et al. (2009) was confounded by the presence of residue retention in the experimental treatment, Liebig et al. (2004) was confounded by a second crop of winter wheat in the experimental treatment, and Bruce et al. (1992) was confounded by a different tillage system in the control (no-till plus a cover crop versus conventional tillage no cover crop) | | | | |
| ^The pasture-based comparisons in the crop and livestock category (3 out of 7 experiments) differed from the two perennial experiments and one crop rotation experiment with livestock because they included a pasture-based control with no livestock and did not represent any changes to crop patterns (annual versus perennial, monoculture versus crop rotation). | | | | |

**Additional database considerations**

There were a few studies that represented more complex experimental designs that measured subsamples from larger areas rather than taking independent samples from randomized block designs. When these studies did not explicitly describe a true replication method, or if they did not report the number of replicates in the experiment, these studies were assigned a replication value of 1, which would ascribe a lower weight in the statistical calculations for these experiments. Those studies were:

- Arshad et al. 1999 (no-till): Replication number not reported
- Nyamadzawo et al. 2003, 2008 (crop rotation & no-till): Replication number not reported
- TerAvest et al. 2015 (crop rotation & no-till): Sampled from adjacent fields
- Proffitt et al. 1995 (crop and livestock): Sampled from adjacent fields
- Ketema and Yimer 2014 (perennial): Sampled from adjacent fields (replication reported)

We focused on field experiments that were developed to test for effects of individual practices on infiltration rates but after careful consideration, we also included six experiments that met all other selection criteria but where designs varied such that treatment effects were confounded by one factor (e.g. conventional tillage compared to no-till plus mulch, conventional tillage and no cover crop compared to no-till plus a cover crop, monoculture crop compared to crop rotation plus livestock; noted in Table 1).

When an experiment included more than one treatment that could be considered a control (e.g. crop rotation experiments that included more than one monoculture), these observations were averaged to create a single control for each of the appropriate treatment measurements (4 of 89 experiments; S1 Table 1). By averaging the controls in this limited number of studies, we were able to attain an estimate for the likely broader effect of the practice of interest on the conventional treatment, while recognizing that control conditions can vary.

**Model Selection and R Code**

We used the lme4 package in R as described by Koricheva et al. (2013) for the main statistical analysis which included:

Overall means by group: datasets for each group were analyzed separately

lmer(Li ~ 1 + (1|Study), data=till, weights = Wi)

(1|Study) represents the random effect of study

Wi represents the weighting factor as described in the main text, Equation [2]

No-till and Cover Crops additional analysis

lmer(Li ~ FixedEffects + (1|Study), data=till, weights = Wi)

Fixed Effects represent the categories discussed in the main text, Figures 5 & 6 and described in the methods

Continuous variables (regression coefficients in main text Table 3)

lmer(Li ~ ContinuousVariables + (1|Study), data=till, weights = Wi)

**Additional Results**


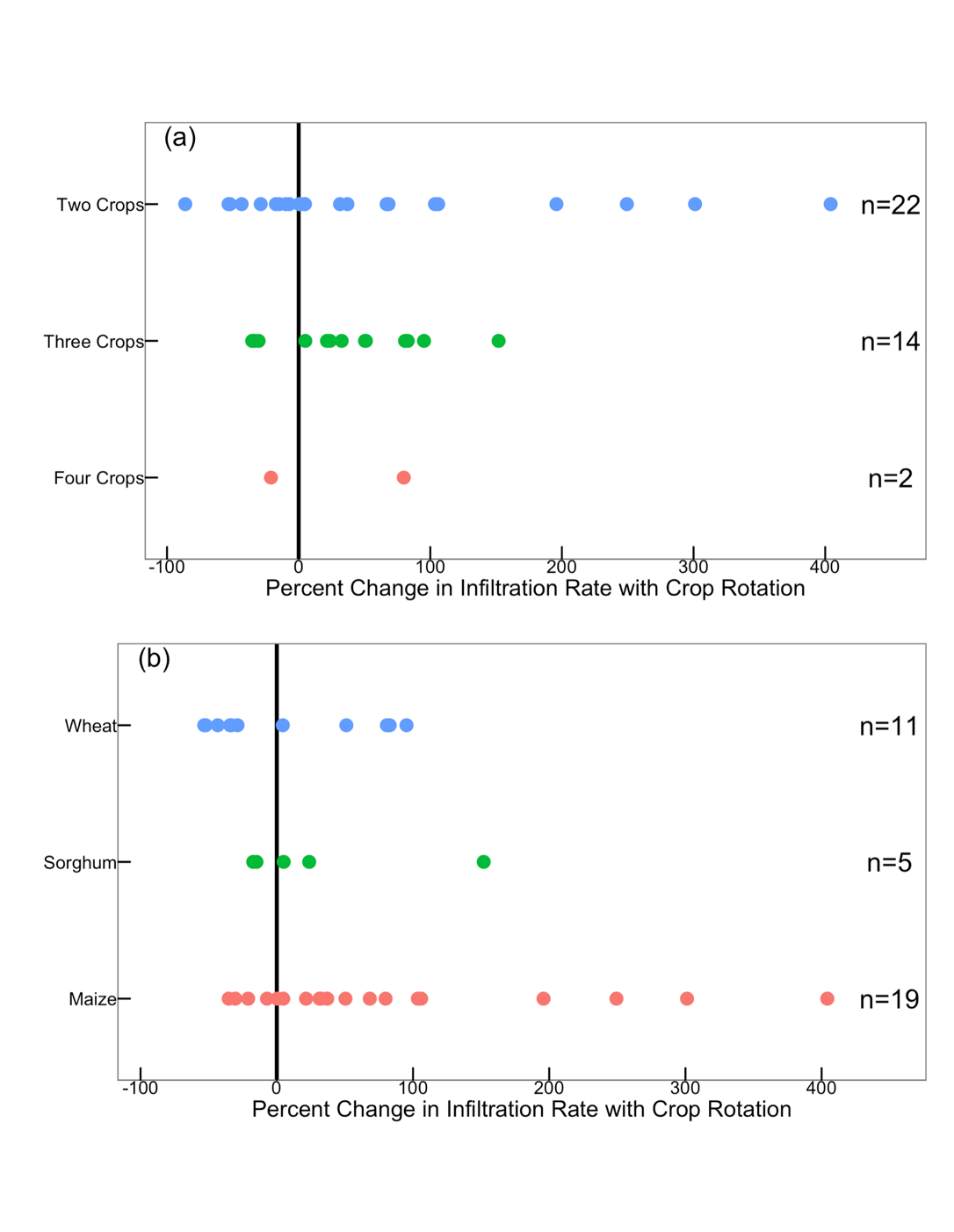


**Fig. A.**  **Response of infiltration rates to sub-categories of crop rotation experiments** (n=number of paired comparisons per group)**.** (a) Paired comparisons grouped by the number of crops included in the experimental treatment rotation. (b) Paired comparisons grouped by the main crop in the control treatment. Note that this does not include Govaerts et al. (2007) which reported maize monoculture and wheat monoculture separately.


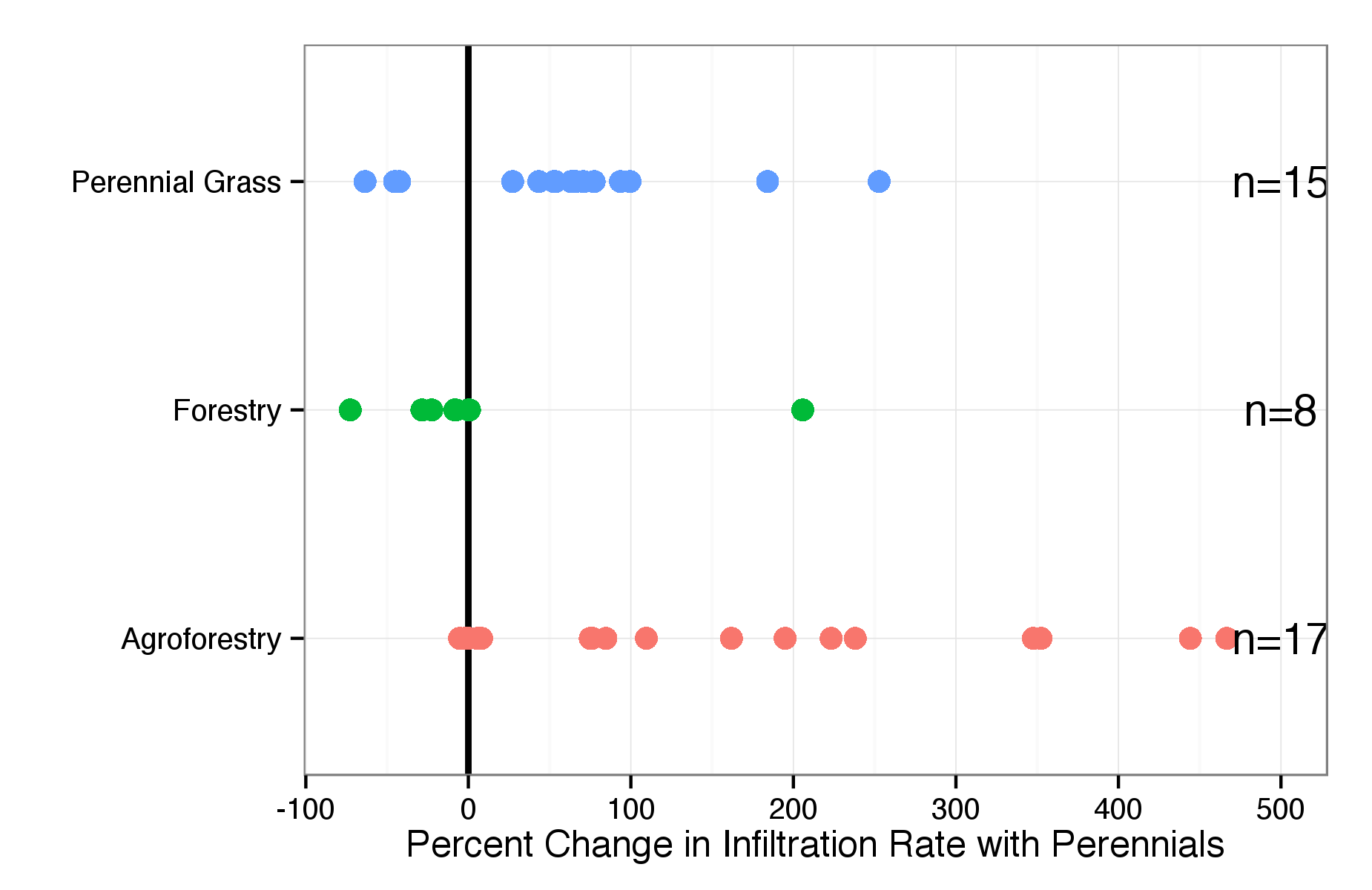


**Fig. B.** **Percent change in infiltration rates for perennial experiments.** Paired comparisons grouped by the three types of included treatments: agroforestry, perennial grasses, and managed forestry (n=number of paired comparisons per group)**.**


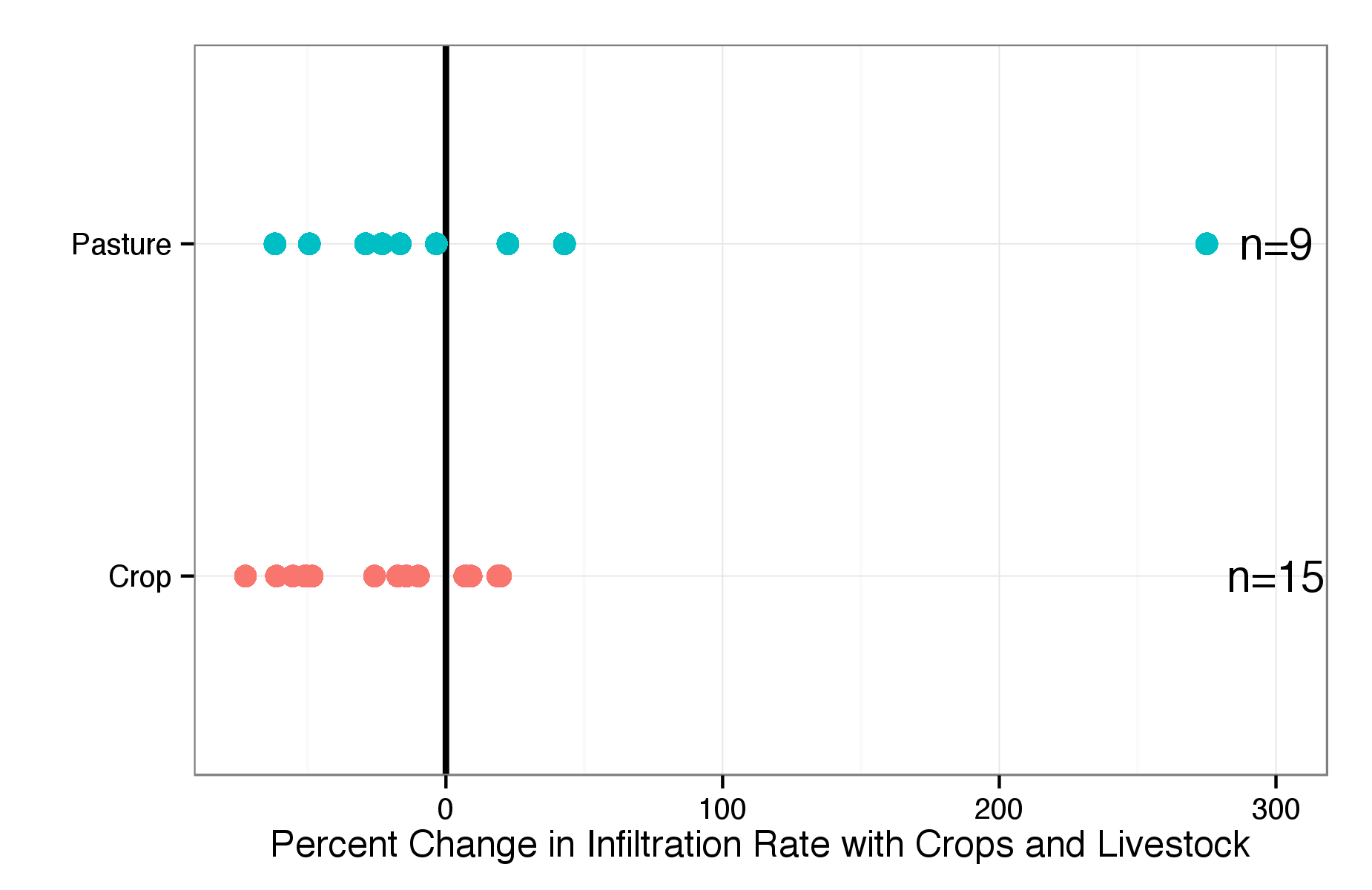


**Fig. C.** **Response of infiltration rates for crop and livestock (cropland grazing) experiments.** Paired comparisons grouped by the presence of annual crops or pasture in control and experimental treatments (n=number of paired comparisons per group)**.**

**(1)**


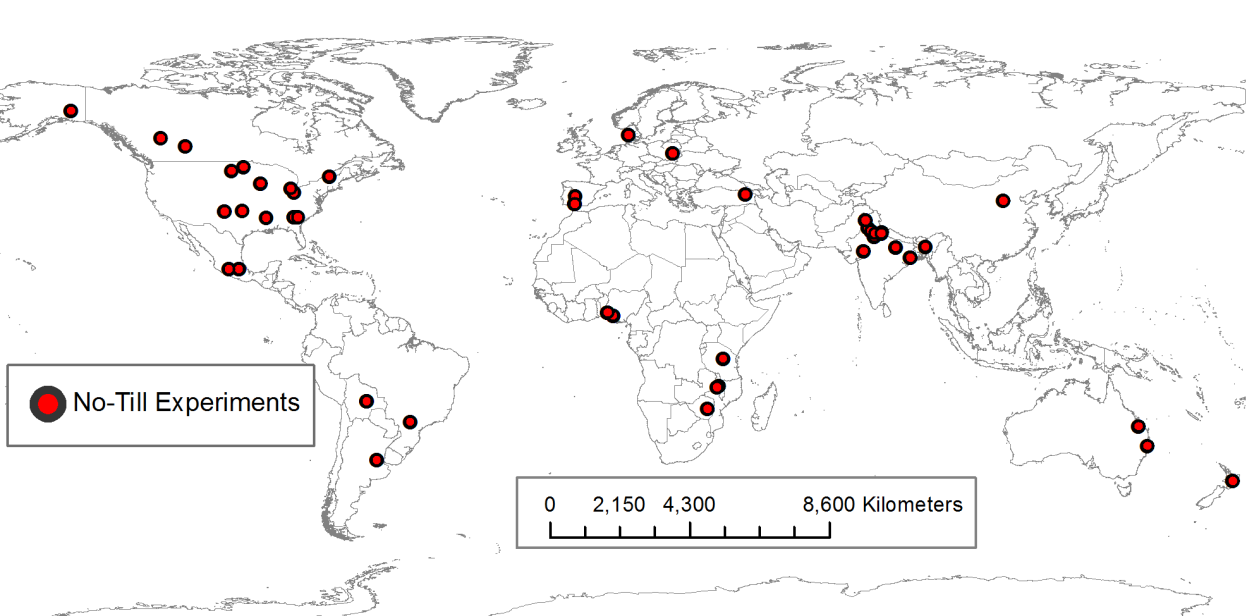


**(2)**


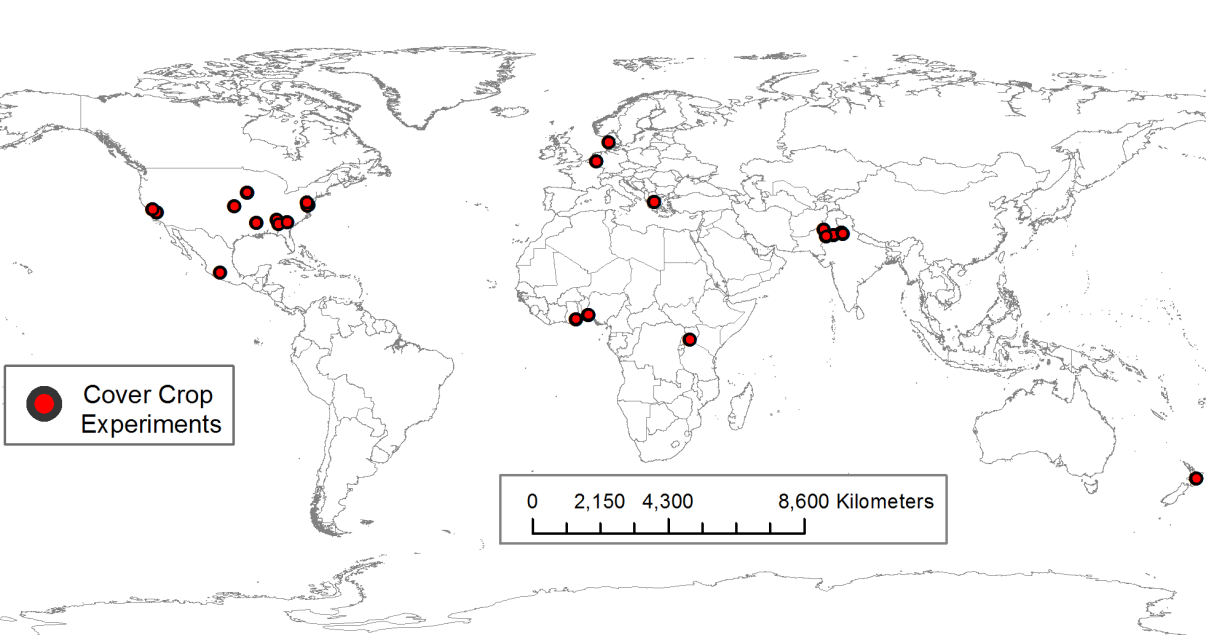


**(3)**


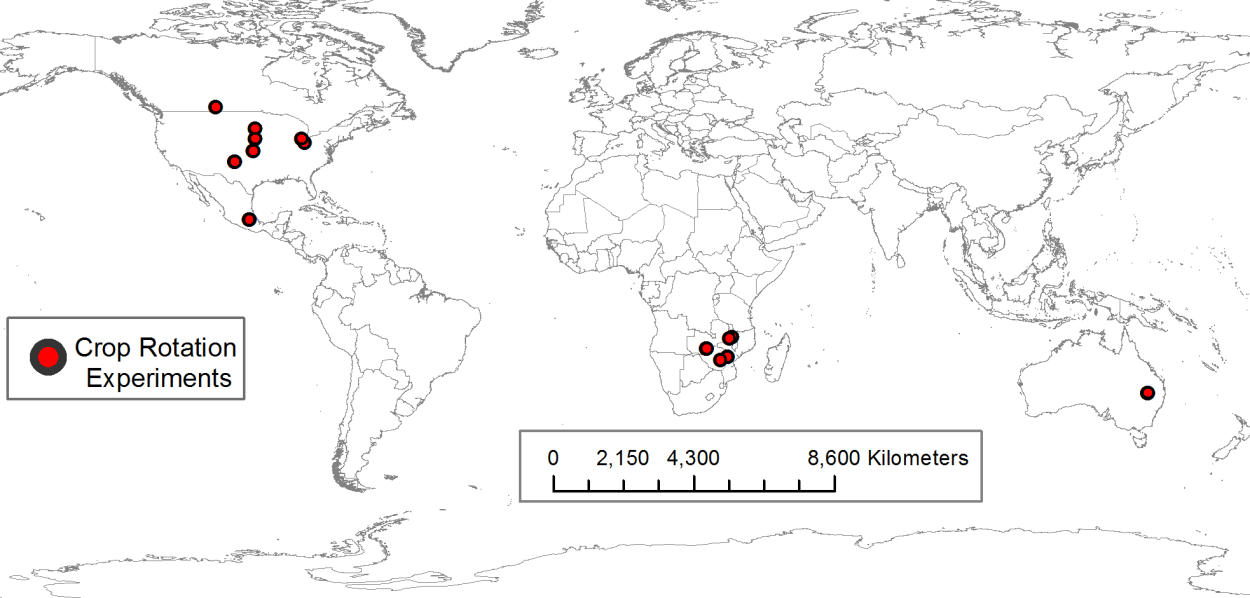


**(4)**


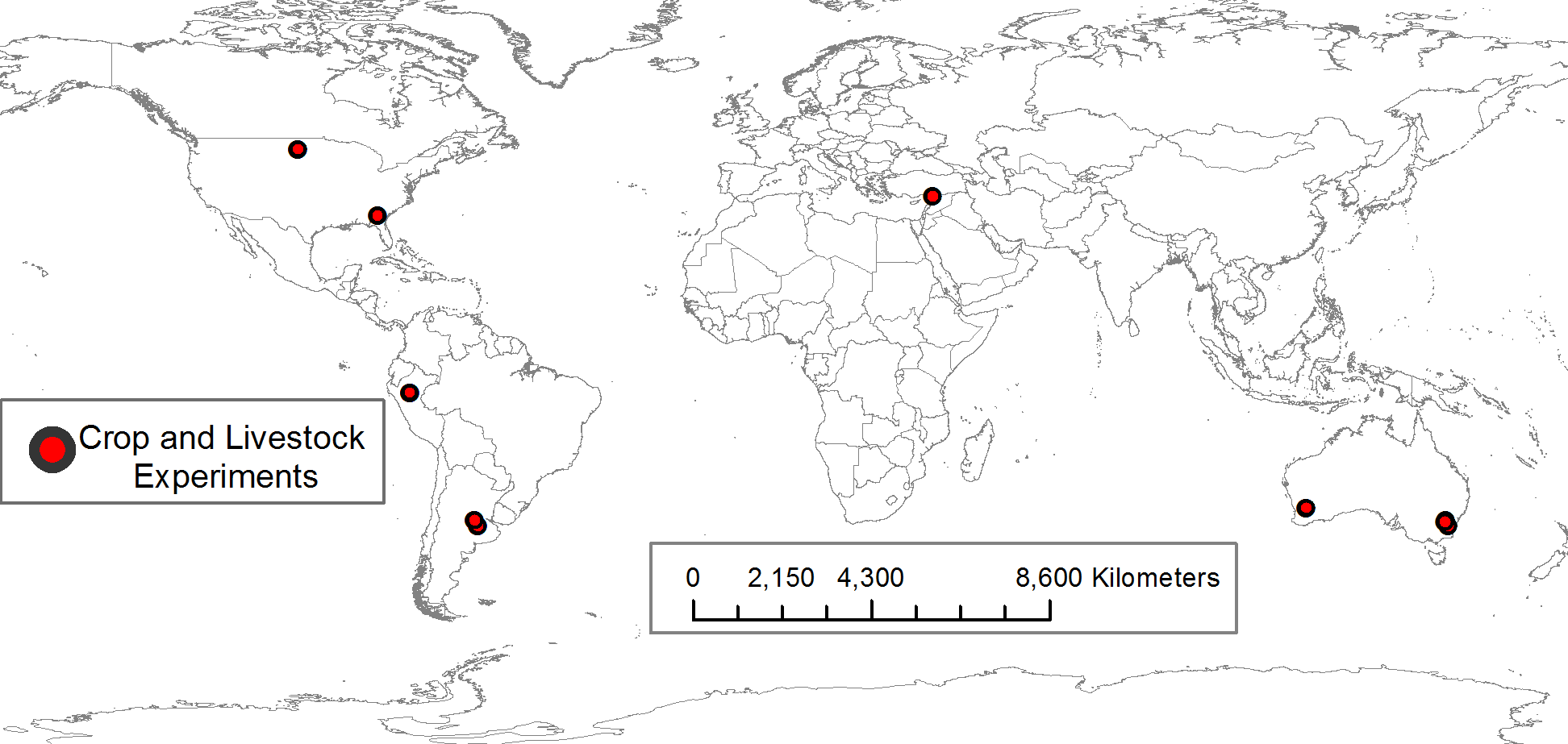


**(5)**


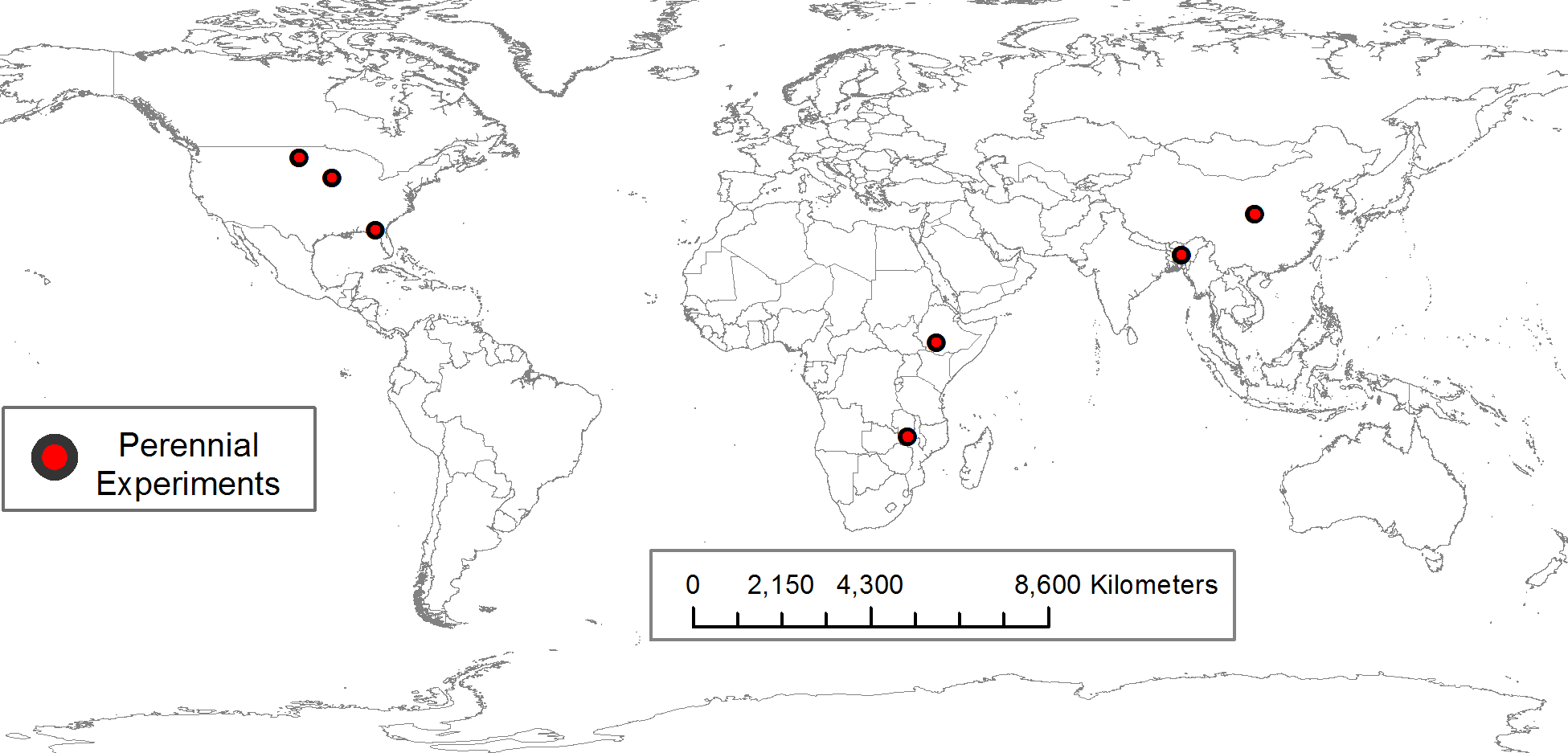


**Fig. D. Experiment locations for each of the different agricultural practices included in the analysis. (1) no-till, (2) cover crops, (3) crop rotation, (4) crop and livestock, (5) perennials.** Maps generated with ESRI ArcGIS ver. 10.4 (<http://www.esri.com>).


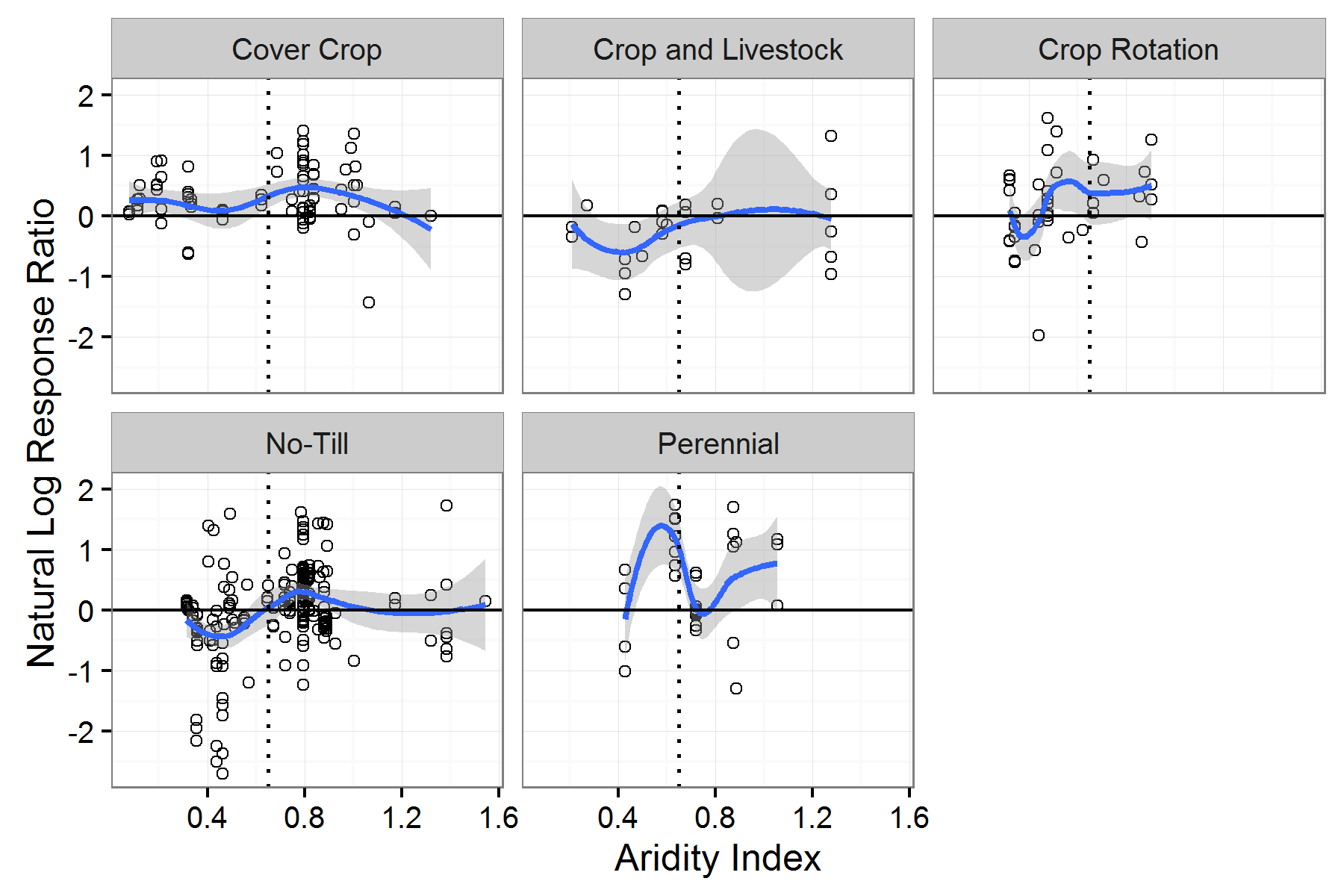


**Fig. E. Natural log response ratio by practice across the range of aridity indices.** Response ratios above zero (solid line) represent a positive effect of the practices on infiltration rates. Smoothed means are represented in blue and gray. Aridity index values greater than 0.65 (dashed line) represent regions considered to have more humid climates.

**(1)**


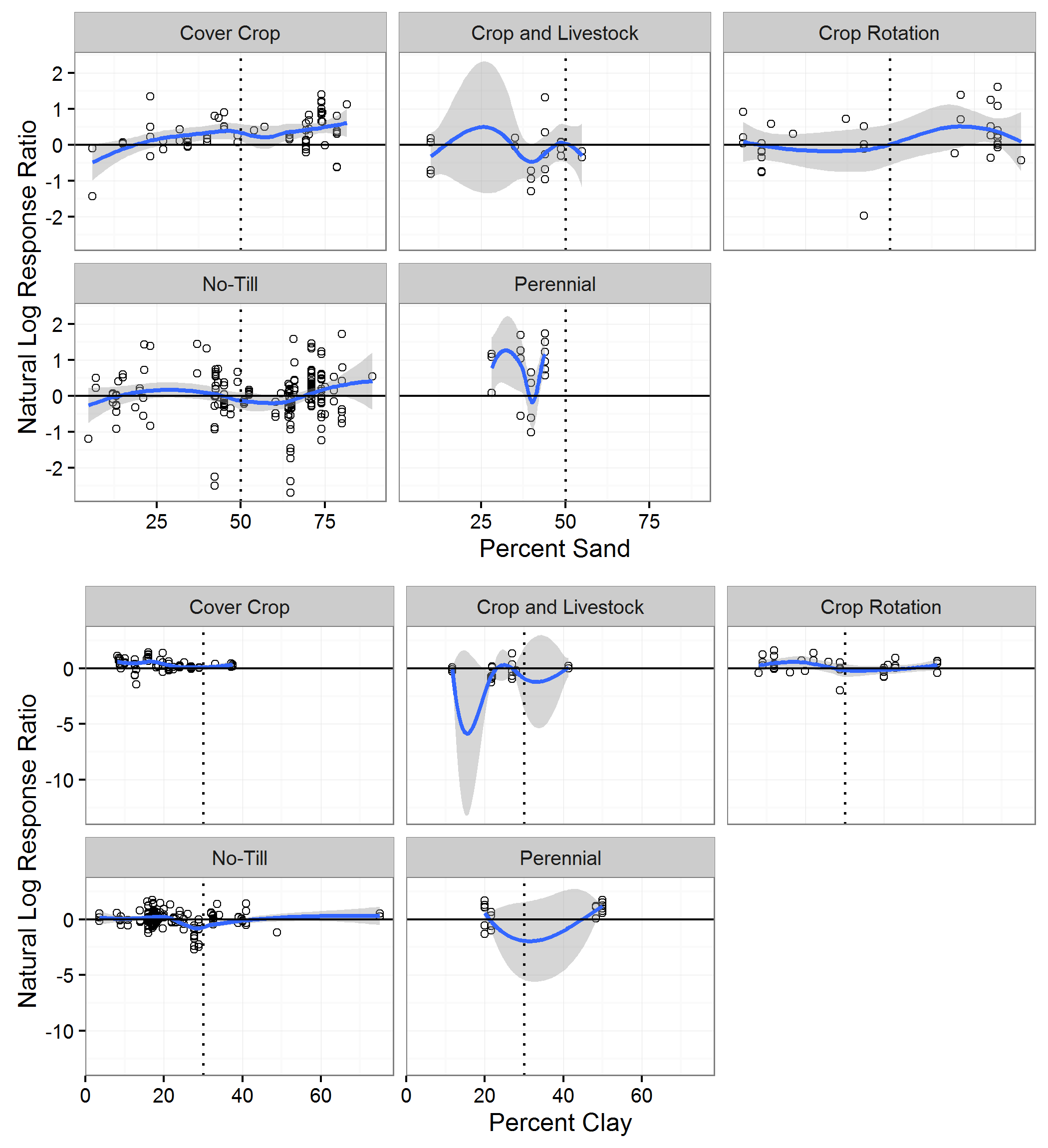


**(2)**


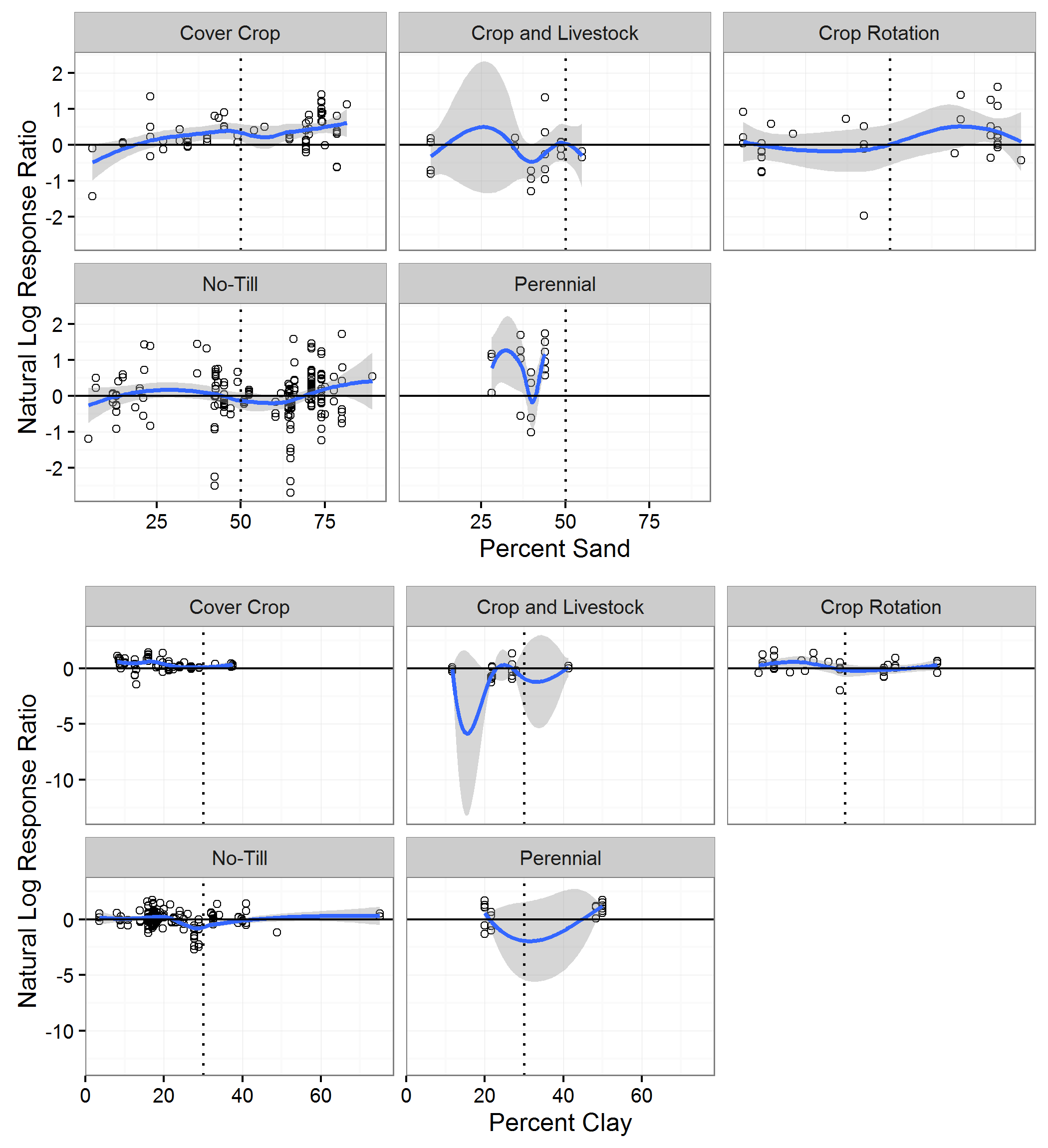


**Fig. F.** **Natural log response ratio by practice category across the range of (1) sand and (2) clay contents.** Smoothed means are represented in blue and gray. Dashed lines represent the broad groups of sand and clay (>50% sand, >30% clay) that were used for the fixed effects analysis in the cover crop and no-till experiments. As a result of data limitations, this figure does not represent some experiments from each category (no-till: 8/52 missing experiments (15%), cover crops: 3/23 (13%), crop rotations: 1/11 (9%), perennials: 2/8 (25%), and cropland grazing: 2/7 (29%).


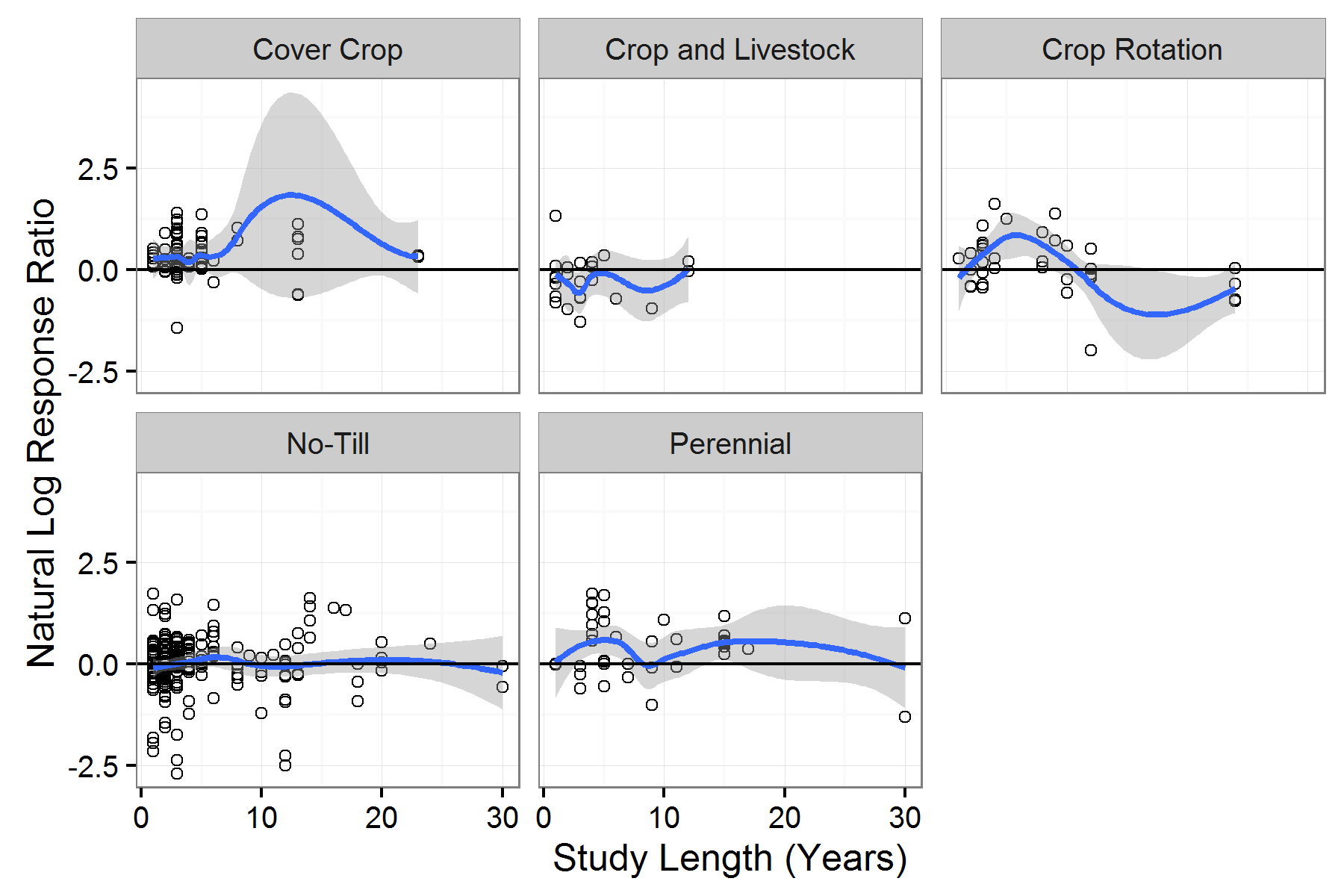


**Fig. G.** **Natural log response ratio by practice category for the effect of study length.** Smoothed means are represented in blue and gray. Response ratios above zero (solid line) represent a positive effect of the conservation practices.

**PRISMA Checklist**

| **Section/topic** | **#** | **Checklist item** | **Reported on page #** |
| --- | --- | --- | --- |
| **TITLE** | | |  |
| Title | 1 | Identify the report as a systematic review, meta-analysis, or both. | Title page: 1 |
| **ABSTRACT** | | |  |
| Structured summary | 2 | Provide a structured summary including, as applicable: background; objectives; data sources; study eligibility criteria, participants, and interventions; study appraisal and synthesis methods; results; limitations; conclusions and implications of key findings; systematic review registration number. | 2 |
| **INTRODUCTION** | | |  |
| Rationale | 3 | Describe the rationale for the review in the context of what is already known. | 3-5 |
| Objectives | 4 | Provide an explicit statement of questions being addressed with reference to participants, interventions, comparisons, outcomes, and study design (PICOS). | 4-5 |
| **METHODS** | | |  |
| Protocol and registration | 5 | Indicate if a review protocol exists, if and where it can be accessed (e.g., Web address), and, if available, provide registration information including registration number. | n/a |
| Eligibility criteria | 6 | Specify study characteristics (e.g., PICOS, length of follow-up) and report characteristics (e.g., years considered, language, publication status) used as criteria for eligibility, giving rationale. | 5-6 |
| Information sources | 7 | Describe all information sources (e.g., databases with dates of coverage, contact with study authors to identify additional studies) in the search and date last searched. | 6-7 |
| Search | 8 | Present full electronic search strategy for at least one database, including any limits used, such that it could be repeated. | 5-6 |
| Study selection | 9 | State the process for selecting studies (i.e., screening, eligibility, included in systematic review, and, if applicable, included in the meta-analysis). | 5-7, PRISMA flow chart, Supp. Info |
| Data collection process | 10 | Describe method of data extraction from reports (e.g., piloted forms, independently, in duplicate) and any processes for obtaining and confirming data from investigators. | 9-10, Supp. Info |
| Data items | 11 | List and define all variables for which data were sought (e.g., PICOS, funding sources) and any assumptions and simplifications made. | 9-10, Supp. Info |
| Risk of bias in individual studies | 12 | Describe methods used for assessing risk of bias of individual studies (including specification of whether this was done at the study or outcome level), and how this information is to be used in any data synthesis. | 11, 16-17 |
| Summary measures | 13 | State the principal summary measures (e.g., risk ratio, difference in means). | 10-11 |
| Synthesis of results | 14 | Describe the methods of handling data and combining results of studies, if done, including measures of consistency (e.g., I^2^) for each meta-analysis. | 7-9, Supp. Info |

| **Section/topic** | **#** | **Checklist item** | **Reported on page #** |
| --- | --- | --- | --- |
| Risk of bias across studies | 15 | Specify any assessment of risk of bias that may affect the cumulative evidence (e.g., publication bias, selective reporting within studies). | 11, 16-17 |
| Additional analyses | 16 | Describe methods of additional analyses (e.g., sensitivity or subgroup analyses, meta-regression), if done, indicating which were pre-specified. | 11, 16-17 |
| **RESULTS** | | |  |
| Study selection | 17 | Give numbers of studies screened, assessed for eligibility, and included in the review, with reasons for exclusions at each stage, ideally with a flow diagram. | Fig 1, 6 |
| Study characteristics | 18 | For each study, present characteristics for which data were extracted (e.g., study size, PICOS, follow-up period) and provide the citations. | 9-10, Supp. Info |
| Risk of bias within studies | 19 | Present data on risk of bias of each study and, if available, any outcome level assessment (see item 12). | 11, 16-17 |
| Results of individual studies | 20 | For all outcomes considered (benefits or harms), present, for each study: (a) simple summary data for each intervention group (b) effect estimates and confidence intervals, ideally with a forest plot. | Fig. 4-6, Supp. Info |
| Synthesis of results | 21 | Present results of each meta-analysis done, including confidence intervals and measures of consistency. | Figs 4-6 |
| Risk of bias across studies | 22 | Present results of any assessment of risk of bias across studies (see Item 15). | 16-17, Figs 8-9 |
| Additional analysis | 23 | Give results of additional analyses, if done (e.g., sensitivity or subgroup analyses, meta-regression [see Item 16]). | 16-17, Figs 8-9 |
| **DISCUSSION** | | |  |
| Summary of evidence | 24 | Summarize the main findings including the strength of evidence for each main outcome; consider their relevance to key groups (e.g., healthcare providers, users, and policy makers). | 12-17 |
| Limitations | 25 | Discuss limitations at study and outcome level (e.g., risk of bias), and at review-level (e.g., incomplete retrieval of identified research, reporting bias). | 22-24 |
| Conclusions | 26 | Provide a general interpretation of the results in the context of other evidence, and implications for future research. | 18-24 |
| **FUNDING** | | |  |
| Funding | 27 | Describe sources of funding for the systematic review and other support (e.g., supply of data); role of funders for the systematic review. | Included in article submission |

**References: Supporting Information**

Arshad MA, Franzluebbers AJ, Azooz RH. Components of surface soil structure under conventional and no-tillage in northwestern Canada. Soil and Tillage Research. 1999, 53: 41-47. http://dx.doi.org/10.1016/S0167-1987(99)00075-6

Ketema H, Yimer F. Soil property variation under agroforestry based conservation tillage and maize based conventional tillage in Southern Ethiopia. Soil and Tillage Research. 2014, 141: 25-31. http://dx.doi.org/10.1016/j.still.2014.03.011

Koricheva J, Gurevitch J, Mengersen K, editors. Handbook of meta-analysis in ecology and evolution. Princeton University Press, 2013.

Nyamadzawo G, Nyamugafata P, Chikowo R, Giller KE. Partitioning of simulated rainfall in a kaolinitic soil under improved fallow–maize rotation in Zimbabwe. Agroforestry Systems. 2013, 59: 207-214. 10.1023/B:AGFO.0000005221.67367.fd

Nyamadzawo G, Nyamugafata P, Chikowo R, Giller K. Residual effects of fallows on selected soil hydraulic properties in a kaolinitic soil subjected to conventional tillage (CT) and no tillage (NT). Agroforestry Systems. 2008, 72: 161-168.10.1007/s10457-007-9057-6

Proffitt AP, Bendotti S, McGarry D. A comparison between continuous and controlled grazing on a red duplex soil. I. Effects on soil physical characteristics. Soil and Tillage Research. 1995, 35: 199-210. doi:10.1016/0167-1987(95)00486-6

Rosenberg M, Adams D, Gurevitch J. MetaWin: Statistical Software for Meta-Analysis. Version 2.0. 2000.

TerAvest D, Carpenter-Boggs L, Thierfelder C, Reganold JP. Crop production and soil water management in conservation agriculture, no-till, and conventional tillage systems in Malawi. Agriculture, Ecosystems & Environment. 2015, 212: 285-96. http://dx.doi.org/10.1016/j.agee.2015.07.011

***Experiments included in the database, Supporting Information Table A***

Abdollahi L, Munkholm LJ. Tillage system and cover crop effects on soil quality: I. Chemical, mechanical, and biological properties. Soil Science Society of America Journal, 2014, 78, 262-70. doi:10.2136/sssaj2013.07.0302

Alemu G, Unger PW, Jones OR. Tillage and cropping system effects on selected conditions of a soil cropped to grain sorghum for twelve years. Communications in Soil Science & Plant Analysis, 1997, 28, 63-71. 10.1080/00103629709369772

Arevalo LA, Alegre JC, Bandy DE, Szott LT. The effect of cattle grazing on soil physical and chemical properties in a silvopastoral system in the Peruvian Amazon. Agroforesty System, 1998, 40, 109-24. 10.1023/A:1006075114659

Arshad MA, Franzluebbers AJ, Azooz RH. Components of surface soil structure under conventional and no-tillage in northwestern Canada. Soil and Tillage Research, 1999, 53, 41-47. http://dx.doi.org/10.1016/S0167-1987(99)00075-6

Astier M, Maass JM, Etchevers-Barra JD, Pena JJ, de León González F. Short-term green manure and tillage management effects on maize yield and soil quality in an Andisol. Soil and Tillage Research, 2006, 88,153-159. 10.1016/j.still.2005.05.003

Bajpai RK, Tripathi RP. Evaluation of non-puddling under shallow water tables and alternative tillage methods on soil and crop parameters in a rice–wheat system in Uttar Pradesh. Soil and Tillage Research, 2000, 55, 99-106. doi:10.1016/S0167-1987(00)00111-2

Barber RG, Orellana M, Navarro F, Diaz O, Soruco MA. Effects of conservation and conventional tillage systems after land clearing on soil properties and crop yield in Santa Cruz, Bolivia. Soil and Tillage Research, 1996, 38: 133-52. doi:10.1016/0167-1987(96)01012-4

Baumhardt RL, Jones OR (2002) Residue management and paratillage effects on some soil properties and rain infiltration. Soil and Tillage Research, 2002, 65, 19-27. 10.1016/S0167-1987(01)00273-2

Baumhardt RL, Johnson GL, Schwartz RC. Residue and long-term tillage and crop rotation effects on simulated rain infiltration and sediment transport. Soil Science Society of America Journal, 2012, 76: 1370-1378. doi:10.2136/sssaj2011.0331

Bazaya BR, Sen A, Srivastava VK. Planting methods and nitrogen effects on crop yield and soil quality under direct seeded rice in the Indo-Gangetic plains of eastern India. Soil and Tillage Research, 2009, 105: 27-32. doi:10.1016/j.still.2009.05.006

Bell LW, Kirkegaard JA, Swan A, Hunt JR, Huth NI, Fettell NA. Impacts of soil damage by grazing livestock on crop productivity. Soil and Tillage Research, 2011, 113: 19-29. 10.1016/j.still.2011.02.003

Bharati L, Lee KH, Isenhart TM, Schultz RC. Soil-water infiltration under crops, pasture, and established riparian buffer in Midwestern USA. Agroforestry Systems, 2002, 56: 249-257. doi:10.1023/A:1021344807285

Bhattacharyya R, Kundu S, Pandey SC, Singh KP, Gupta HS. Tillage and irrigation effects on crop yields and soil properties under the rice–wheat system in the Indian Himalayas. Agricultural Water Management, 2008, 95: 993-1002. 10.1016/j.agwat.2008.03.007

Blanco-Canqui H, Francis CA. Building resilient soils through agroecosystem redesign under fluctuating climatic regimes. Journal of Soil and Water Conservation, 2016, 71: 127A-33A. doi: 10.2489/jswc.71.6.127A

Blanco-Canqui H, Mikha MM, Presley DR, Claassen MM. Addition of cover crops enhances no-till potential for improving soil physical properties. Soil Science Society of America Journal, 2011, 75: 1471-82. doi:10.2136/sssaj2010.0430

Bruce RR, Langdale GW, Dillard AL. Tillage and crop rotation effect on characteristics of a sandy surface soil. Soil Science Society of America Journal, 1990, 54: 1744-1747. doi:10.2136/sssaj1990.03615995005400060039x

Bruce RR, Langdale GW, West LT, Miller WP. Soil surface modification by biomass inputs affecting rainfall infiltration. Soil Science Society of America Journal, 1992, 56: 1614-1620. 10.2136/sssaj1992.03615995005600050046x

Chirwa TS, Mafongoya PL, Chintu R. Mixed planted-fallows using coppicing and non-coppicing tree species for degraded Acrisols in eastern Zambia. Agroforestry Systems, 2003, 59: 243-251. 10.1023/B:AGFO.0000005225.12629.61

Dao TH. Tillage and winter wheat residue management effects on water infiltration and storage. Soil Science Society of America Journal, 1993, 57: 1586-1595. 10.2136/sssaj1993.03615995005700060032x

de Moraes MT, Debiasi H, Carlesso R, Franchini JC, da Silva VR, da Luz FB. Soil physical quality on tillage and cropping systems after two decades in the subtropical region of Brazil. Soil and Tillage Research, 2016, 155: 351-362. 10.1016/j.still.2015.07.015

Fernández PL, Alvarez CR, Taboada MA. Topsoil compaction and recovery in integrated no-tilled crop–livestock systems of Argentina. Soil and Tillage Research, 2015, 153: 86-94. http://dx.doi.org/10.1016/j.still.2015.05.008

Fischler M, Wortmann CS, Feil B. Crotalaria (C. ochroleuca G. Don.) as a green manure in maize–bean cropping systems in Uganda. Field Crops Research, 1999, 61: 97-107. 10.1016/S0378-4290(98)00150-6

Folorunso OA, Rolston DE, Prichard T, Loui DT. Soil surface strength and infiltration rate as affected by winter cover crops. Soil Technology, 1992, 5: 189-197. 10.1016/0933-3630(92)90021-R

Franzen H, Lal R, Ehlers W. Tillage and mulching effects on physical properties of a tropical Alfisol. Soil and Tillage Research, 1994, 28: 329-346. doi:10.1016/0167-1987(94)90139-2

Franzluebbers AJ, Stuedemann JA. Soil physical responses to cattle grazing cover crops under conventional and no tillage in the Southern Piedmont USA. Soil and Tillage Research, 2008, 100: 141-153. 10.1016/j.still.2008.05.011

Franzluebbers AJ, Stuedemann JA, Franklin DH. Water infiltration and surface-soil structural properties as influenced by animal traffic in the Southern Piedmont USA. Renewable Agriculture and Food Systems, 2012, 27: 256-265. 10.1017/S1742170511000378

Gangwar KS, Singh KK, Sharma SK, Tomar OK. Alternative tillage and crop residue management in wheat after rice in sandy loam soils of Indo-Gangetic plains. Soil and Tillage Research, 2006, 88: 242-252. doi:10.1016/j.still.2005.06.015

Ghafoor A, Murtaza G, Rehman MZ, Sabir M. Reclamation and salt leaching efficiency for tile drained saline‐sodic soil using marginal quality water for irrigating rice and wheat crops. Land Degradation and Development, 2012, 23(1): 1-9. 10.1002/ldr.1033

Ghosh PK, Saha R, Gupta JJ, Ramesh T, Das A, Lama TD, Munda GC, Bordoloi JS, Verma MR, Ngachan SV. Long-term effect of pastures on soil quality in acid soil of North-East India. Soil Research, 2009, 47: 372-379. doi:10.1071/SR08169

Ghuman BS, Lal R. Effects of soil wetness at the time of land clearing on physical properties and crop response on an Ultisol in southern Nigeria. Soil and Tillage Research, 1992, 22: 1-11. doi:10.1016/0167-1987(92)90018-7

Gómez-Paccard C, Hontoria C, Mariscal-Sancho I, Pérez J, León P, González P, Espejo R. Soil–water relationships in the upper soil layer in a Mediterranean Palexerult as affected by no-tillage under excess water conditions–Influence on crop yield. Soil and Tillage Research, 2015, 146: 303-312. http://dx.doi.org/10.1016/j.still.2014.09.012

Govaerts, B., Verhulst, N., Castellanos-Navarrete, A., Sayre, K.D., Dixon, J., Dendooven, L. Conservation Agriculture and Soil Carbon Sequestration: Between Myth and Farmer Reality. Critical Reviews in Plant Sciences. 2009, 28: 97–122. doi:10.1080/07352680902776358

Gozubuyuk Z, Sahin U, Ozturk I, Celik A, Adiguzel MC. Tillage effects on certain physical and hydraulic properties of a loamy soil under a crop rotation in a semi-arid region with a cool climate. Catena, 2014, 118: 195-205. 10.1016/j.catena.2014.01.006

Gulick, S.H., Grimes, D.W., Goldhamer, D.A. and Munk, D.S. Cover-crop-enhanced water infiltration of a slowly permeable fine sandy loam. Soil Science Society of America Journal, 1994, 58:1539-1546. 10.2136/sssaj1994.03615995005800050038x

Guzha AC. Effects of tillage on soil microrelief, surface depression storage and soil water storage. Soil and Tillage Research, 2004, 76: 105-114. 10.1016/j.still.2003.09.002

He J, Wang Q, Li H, Tullberg JN, McHugh AD, Bai Y, Zhang X, McLaughlin N, Gao H. Soil physical properties and infiltration after long‐term no‐tillage and ploughing on the Chinese Loess Plateau. New Zealand Journal of Crop and Horticultural, 2009, 37: 157-166. http://dx.doi.org/10.1080/01140670909510261

Jat ML, Gathala MK, Saharawat YS, Tetarwal JP, Gupta R. Double no-till and permanent raised beds in maize–wheat rotation of north-western Indo-Gangetic plains of India: Effects on crop yields, water productivity, profitability and soil physical properties. Field Crops Research, 2013, 149: 291-299. 10.1016/j.fcr.2013.04.024

Kahlown MA, Azam M. Effect of saline drainage effluent on soil health and crop yield. Agricultural Water Management, 2003, 62: 127-138. 10.1016/S0378-3774(03)00096-9

Kaspar TC, Radke JK, Laflen JM. Small grain cover crops and wheel traffic effects on infiltration, runoff, and erosion. Journal of Soil and Water Conservation, 2001, 56: 160-164.

Kayombo B, Lal R, Mrema GC, Jensen HE. Characterizing compaction effects on soil properties and crop growth in southern Nigeria. Soil and Tillage Research, 1991, 21: 325-345. doi:10.1016/0167-1987(91)90029-W

Ketema H, Yimer F. Soil property variation under agroforestry based conservation tillage and maize based conventional tillage in Southern Ethiopia. Soil and Tillage Research, 2014, 141: 25-31. http://dx.doi.org/10.1016/j.still.2014.03.011

Khan AR. Studies on tillage-induced physical edaphic properties in relation to peanut crop. Soil and Tillage Research, 1984, 4: 225-236. doi:10.1016/0167-1987(84)90022-9

Kumar S, Kadono A, Lal R, Dick W. Long-term tillage and crop rotations for 47–49 years influences hydrological properties of two soils in Ohio. Soil Science Society of America Journal, 2012, 76: 2195-2207. doi:10.2136/sssaj2012.0098

Kuotsu K, Das A, Lal R, Munda GC, Ghosh PK, Ngachan SV. Land forming and tillage effects on soil properties and productivity of rainfed groundnut (Arachis hypogaea L.)–rapeseed (Brassica campestris L.) cropping system in northeastern India. Soil and Tillage Research, 2014, 142: 15-24. http://dx.doi.org/10.1016/j.still.2014.04.008

Kwaad FJ, Van Mulligen EJ. Cropping system effects of maize on infiltration, runoff and erosion on loess soils in South-Limbourg (The Netherlands): a comparison of two rainfall events. Soil Technology, 1991, 4: 281-295. doi: 10.1016/0933-3630(91)90007-A

Lal R, Wilson GF, Okigbo BN. No-till farming after various grasses and leguminous cover crops in tropical alfisol. I. Crop performance. Field Crops Research, 1978, 1: 71-84. doi:10.1016/0378-4290(78)90008-4

Lal R. Long-term tillage and maize monoculture effects on a tropical Alfisol in western Nigeria. I. Crop yield and soil physical properties. Soil and Tillage Research, 1997, 42: 145-160. doi:10.1016/S0167-1987(97)00006-8

Lal R, Logan TJ, Fausey NR. Long-term tillage and wheel traffic effects on a poorly drained mollic ochraqualf in northwest Ohio. 2. Infiltrability, surface runoff, sub-surface flow and sediment transport. Soil and Tillage Research, 1989, 14: 359-373. doi:10.1016/0167-1987(89)90055-X

Laddha KC, Totawat KL. Effects of deep tillage under rainfed agriculture on production of sorghum (Sorghum biocolor L. Moench) intercropped with green gram (Vigna radiata L. Wilczek) in western India. Soil and Tillage Research, 1997, 43: 241-250. doi:10.1016/S0167-1987(97)00027-5

Levi MR, Shaw JN, Wood CW, Hermann SM, Carter EA, Feng Y. Land management effects on near-surface soil properties of southeastern US coastal plain Kandiudults. Soil Science Society of America Journal, 2010, 74: 258-271. doi:10.2136/sssaj2009.0015

Liebig MA, Tanaka DL, Wienhold BJ. Tillage and cropping effects on soil quality indicators in the northern Great Plains. Soil and Tillage Research 2004, 78: 131-141. 10.1016/j.still.2004.02.002

Liebig MA, Tanaka DL, Kronberg SL, Scholljegerdes EJ, Karn JF. Soil hydrological attributes of an integrated crop-livestock agroecosystem: Increased adaptation through resistance to soil change. Applied and Environmental Soil Science, 2011, 1-6. http://dx.doi.org/10.1155/2011/464827

Lipiec J, Kuś J, Słowińska-Jurkiewicz A, Nosalewicz A. Soil porosity and water infiltration as influenced by tillage methods. Soil and Tillage Research, 2006, 89: 210-220. 10.1016/j.still.2005.07.012

Locke MA, Zablotowicz RM, Steinriede RW, Testa S, Reddy KN. Conservation management in cotton production: Long-term soil biological, chemical, and physical changes. Soil Science Society of America Journal, 2003, 77: 974-984. 10.2136/sssaj2012.0325

Logsdon SD, Jordahl JL, Karlen DL. Tillage and crop effects on ponded and tension infiltration rates. Soil and Tillage Research, 1993, 28: 179-89. doi:10.1016/0167-1987(93)90025-K

Mahmood-ul-Hassan M, Rafique E, Rashid A. Physical and hydraulic properties of aridisols as affected by nutrient and crop-residue management in a cotton-wheat system. Acta Scientiarum. Agronomy, 2013, 35: 127-137. doi: 10.4025/actasciagron.v35i1.14683

Masri Z, Ryan J. Soil organic matter and related physical properties in a Mediterranean wheat-based rotation trial. Soil and Tillage Research, 2006, 87: 146-154. 10.1016/j.still.2005.03.003

McVay KA, Radcliffe DE, Hargrove WL (1989) Winter legume effects on soil properties and nitrogen fertilizer requirements. Soil Science Society of America Journal, 53, 1856-1862. 10.2136/sssaj1989.03615995005300060040x

Moebius-Clune BN, van Es HM, Idowu OJ, Schindelbeck RR, Moebius-Clune DJ, Wolfe DW, Abawi GS, Thies JE, Gugino BK, Lucey R. Long-term effects of harvesting maize stover and tillage on soil quality. Soil Science Society of America Journal, 2008, 72: 960-969. doi:10.2136/sssaj2007.0248

Naresh RK, Tomar SS, Kumar D, Sing S, Dwivedi A, Kumar V. Experiences with rice grown on permanent raised beds: effect of crop establishment techniques on water use, productivity, profitability and soil physical properties. Rice Science, 2014, 21: 170-180. doi:10.1016/S1672-6308(13)60185-7

Nyalemegbe KK, Asiedu EK, Ampontuah EO, Nyamekye AL, Danso SK. Improving the productivity of vertisols in the Accra plains of Ghana using leguminous cover crops. International Journal of Agricultural Sustainability, 2011, 9: 434-442. 10.1080/14735903.2011.583480

Nyamadzawo G, Nyamugafata P, Chikowo R, Giller KE. Partitioning of simulated rainfall in a kaolinitic soil under improved fallow–maize rotation in Zimbabwe. Agroforestry Systems, 2003, 59: 207-214. 10.1023/B:AGFO.0000005221.67367.fd

Nyamadzawo G, Nyamugafata P, Chikowo R, Giller K. Residual effects of fallows on selected soil hydraulic properties in a kaolinitic soil subjected to conventional tillage (CT) and no tillage (NT). Agroforestry Systems, 2008, 72: 161-168.10.1007/s10457-007-9057-6

Pelegrín F, Moreno F, Martin-Aranda J, Camps M. The influence of tillage methods on soil physical properties and water balance for a typical crop rotation in SW Spain. Soil and Tillage Research, 1990, 16: 345-358. doi:10.1016/0167-1987(90)90070-T

Pikul JL, Schwartz RC, Benjamin JG, Baumhardt RL, Merrill S. Cropping system influences on soil physical properties in the Great Plains. Renewable Agriculture and Food Systems, 2006, 21: 15-25. doi:10.1079/RAF2005122

Proffitt AP, Bendotti S, McGarry D. A comparison between continuous and controlled grazing on a red duplex soil. I. Effects on soil physical characteristics. Soil and Tillage Research, 1995, 35: 199-210. doi:10.1016/0167-1987(95)00486-6

Ram H, Singh Y, Saini KS, Kler DS, Timsina J. Tillage and planting methods effects on yield, water use efficiency and profitability of soybean–wheat system on a loamy sand soil. Experimental Agriculture, 2013, 49: 524-542. doi:10.1017/S0014479713000264

Rusinamhodzi L, Corbeels M, Nyamangara J, Giller KE. Maize–grain legume intercropping is an attractive option for ecological intensification that reduces climatic risk for smallholder farmers in central Mozambique. Field Crops Research, 2012, 136: 12-22. 10.1016/j.fcr.2012.07.014

Sasal MC, Andriulo AE, Taboada MA. Soil porosity characteristics and water movement under zero tillage in silty soils in Argentinian Pampas. Soil and Tillage Research, 2006, 87: 9-18. 10.1016/j.still.2005.02.025

Sharma AR, Singh R, Dhyani SK, Dube RK. Moisture conservation and nitrogen recycling through legume mulching in rainfed maize (Zea mays)–wheat (Triticum aestivum) cropping system. Nutrient Cycling in Agroecosystems, 2010, 87: 187-197. doi:10.1007/s10705-009-9327-y

Sharma P, Abrol V, Sharma RK. Impact of tillage and mulch management on economics, energy requirement and crop performance in maize–wheat rotation in rainfed subhumid inceptisols, India. European Journal of Agronomy, 2011, 34: 46-51. 10.1016/j.eja.2010.10.003

Sharratt B, Zhang M, Sparrow S. Twenty years of tillage research in subarctic Alaska: I. Impact on soil strength, aggregation, roughness, and residue cover. Soil and Tillage Research, 2006, 91: 75-81. 10.1016/j.still.2005.11.006

Singh B, Chanasyk DS, McGill WB. Soil hydraulic properties of an Orthic Black Chernozem under long-term tillage and residue management. Canadian Journal of Soil Science, 1996, 76: 63-71. 10.4141/cjss96-010

Singh G, Jalota SK, Singh Y. Manuring and residue management effects on physical properties of a soil under the rice–wheat system in Punjab, India. Soil and Tillage Research, 2007, 94: 229-238. 10.1016/j.still.2006.07.020

Singh VK, Dwivedi BS, Singh SK, Majumdar K, Jat ML, Mishra RP, Rani M. Soil physical properties, yield trends and economics after five years of conservation agriculture based rice-maize system in north-western India. Soil and Tillage Research, 2016, 155: 133-148. 10.1016/j.still.2015.08.001

So HB, Grabski A, Desborough P. The impact of 14 years of conventional and no-till cultivation on the physical properties and crop yields of a loam soil at Grafton NSW, Australia. Soil and Tillage Research, 2009, 104: 180-184. 10.1016/j.still.2008.10.017

Springett JA, Gray RA, Reid JB. Effect of introducing earthworms into horticultural land previously denuded of earthworms. Soil Biology and Biochemistry, 1992, 24: 1615-1622. 10.1016/0038-0717(92)90159-U

Steele MK, Coale FJ, Hill RL. Winter annual cover crop impacts on no-till soil physical properties and organic matter. Soil Science Society of America Journal, 2012, 76: 2164-2173. 10.2136/sssaj2012.0008

TerAvest D, Carpenter-Boggs L, Thierfelder C, Reganold JP. Crop production and soil water management in conservation agriculture, no-till, and conventional tillage systems in Malawi. Agriculture, Ecosystems & Environment, 2015, 212: 285-96. http://dx.doi.org/10.1016/j.agee.2015.07.011

Terzoudi CB, Gemtos TA, Danalatos NG, Argyrokastritis I. Applicability of an empirical runoff estimation method in central Greece. Soil and Tillage Research, 2007, 92: 198-212. 10.1016/j.still.2006.03.002

Thierfelder C, Wall PC. Rotation in conservation agriculture systems of Zambia: effects on soil quality and water relations. Experimental Agriculture, 2010, 46: 309-325. doi:10.1017/S001447971000030X

Thomas GA, Dalal RC, Weston EJ, Lehane KJ, King AJ, Orange DN, Holmes CJ, Wildermuth GB. Pasture–crop rotations for sustainable production in a wheat and sheep-based farming system on a Vertosol in south-west Queensland, Australia. Animal Production Science, 2009, 49: 682-695. 10.1071/EA07170

Thorburn PJ. Structural and hydrological changes in a Vertisol under different fallow management techniques. Soil and Tillage Research, 1992, 23: 341-359. doi:10.1016/0167-1987(92)90080-U

Tripathi RP, Sharma P, Singh S. Influence of tillage and crop residue on soil physical properties and yields of rice and wheat under shallow water table conditions. Soil and Tillage Research, 2007, 92: 221-226. 10.1016/j.still.2006.03.008

Walia MK, Walia SS, Dhaliwal SS. Long-term effect of integrated nutrient management of properties of Typic Ustochrept after 23 cycles of an irrigated rice (Oryza sativa L.)–wheat (Triticum aestivum L.) system. Journal of Sustainable Agriculture, 2010, 34: 724-743. 10.1080/10440046.2010.507519

Wang L, Zhong C, Gao P, Xi W, Zhang S. Soil Infiltration Characteristics in Agroforestry Systems and Their Relationships with the Temporal Distribution of Rainfall on the Loess Plateau in China. PloS One, 2015, 10(4): e0124767. doi:10.1371/journal.pone.0124767

Wilson GF, Lal R, Okigbo BN. Effects of cover crops on soil structure and on yield of subsequent arable crops grown under strip tillage on an eroded Alfisol. Soil and Tillage Research, 1982, 2: 233-250. doi:10.1016/0167-1987(82)90013-7

Yaduvanshi NP, Sharma DR. Tillage and residual organic manures/chemical amendment effects on soil organic matter and yield of wheat under sodic water irrigation. Soil and Tillage Research, 2008, 98: 11-16. 10.1016/j.still.2007.09.010
